# Titin Serum Level Is Elevated in Patients with Myocarditis Depending on Their Genetic Background

**DOI:** 10.3390/jcdd13100487

**Published:** 2026-09-28

**Authors:** Yuriy Vakhrushev, Sofia Mayrina, Sofiya Andreeva, Yulia Vlasova, Yulia Fomicheva, Darina Sambur, Lubov Mitrofanova, Olga Moiseeva, Anna Kostareva

**Affiliations:** 1 Almazov National Medical Research Centre, Saint Petersburg 197341, Russia; sonya.spbgmu16@gmail.com (S.M.); andreeva.sofi2012@yandex.ru (S.A.); vlasova_yua@almazovcentre.ru (Y.V.); yulya.fomicheva.2015@mail.ru (Y.F.); sambur_db@almazovcentre.ru (D.S.); mitrofanova_lb@almazovcentre.ru (L.M.); moiseeva.cardio@gmail.com (O.M.); akostareva@hotmail.com (A.K.); 2 Department of Women’s and Children’s Health and Center for Molecular Medicine, Karolinska Institute, 17177 Stockholm, Sweden

**Keywords:** myocarditis, cardiomyopathy, titin

## Abstract

Background: The molecular basis of myocarditis is often associated with genetic cardiomyopathies and truncating or loss-of-function variants in the titin gene (*TTN*tv/lof). Here, we hypothesize that titin serum levels increase in patients with myocarditis, and that this can depend on the genetic background of the disease. Methods: Forty-six patients with myocarditis were included in this study. Genotyping was performed using targeted panel sequencing. Serum titin levels and titin/creatinine ratios were measured in a subgroup of 27 patients. Results: Overall, 47 gene variants were identified, including 13 pathogenic and likely pathogenic variants and 34 variants of uncertain significance. The frequency of *TTN*tv/lof in patients with myocarditis was 11%. We detected significant differences between the *TTN*tv/lof group and the genotype-negative group in sustained ventricular tachycardia prevalence, myocardial reverse remodeling (∆ end-diastolic volume) and the prevalence of myocardial necrosis. Serum titin level was elevated in patients with myocarditis, negatively correlated with left ventricular ejection fraction, and was associated with ventricular arrhythmias and a low rate of myocardial reverse remodeling. Conclusions: In patients with biopsy-proven myocarditis, the rate of loss-of-function (LOF) variants was 28%, with a predominance of variants in structural and cytoskeletal genes, and *TTN*tv/lof variants were detected in 11% of patients. *TTN*tv/lof variant carriers were characterized by fewer morphological signs of inflammation and more favorable myocardial reverse remodeling. Serum titin levels were elevated in patients with myocarditis. Genotype-negative status of patients was characterized by the highest serum titin levels and titin/creatinine ratios, a lesser degree of myocardial reverse remodeling, sustained ventricular arrhythmias, and a larger area of necrosis.

## 1. Introduction

The molecular, cellular and morphological nature of myocarditis has transformed greatly over the years, shifting from a predominantly infectious etiology to an immune-mediated paradigm and the acceptance of genetic factors in triggering an immune response. It is now widely agreed that myocardial inflammation can arise in primary genetic cardiomyopathy with indolent, low-grade damage to cardiomyocyte structures and myocardial tissue [1,2]. This damage leads to cardiomyocyte loss, progression of fibrosis, myocardial remodeling and development of cardiomyopathy. Simultaneously, genetically predicted cardiomyocyte damage can trigger the generation of anti-myocardial antibodies and sustain the inflammation. Together, the co-occurrence of genetically determined cardiomyopathy with low-grade, aseptic myocardial inflammation and indolent myocarditis is currently agreed upon. The disease progression arises either from a genetically predicted cardiomyocyte loss or from immune-mediated injury and fibrosis. In real life, these two components commonly coexist, leading to contractile cardiomyocyte dysfunction and the development of heart failure [3]. However, prioritizing between these two mechanisms becomes increasingly important, both for the choice of treatment strategies and for accurate prognosis [4]. In this regard, the search for additional markers of cardiac damage, linking the etiology to treatment and prognosis, is of great clinical importance both in primary cardiac diseases and, especially, in myocarditis.

One of the key genes associated with the development of myocarditis and dilated cardiomyopathy is the titin gene (*TTN*), which encodes the largest protein in the human body, consisting of up to 27,000–30,000 amino acids [5]. Titin is one of the main components of myofibrils in both skeletal muscle cells and cardiomyocytes, which spans from the Z-disk to the M-band of the sarcomere and provides muscle extensibility and elasticity [6,7]. The importance of this giant protein for cardiomyocyte structure and function is evidenced by the fact that *TTN* mutations with loss-of-function mechanism of action (mainly truncations, *TTN*tv/lof) cause up to 30% of genetic forms of dilated cardiomyopathy and can also lead to restrictive, arrhythmogenic cardiomyopathies and left ventricular non-compaction. In 2022, Lota et al. conducted a large-scale genetic study on patients with myocarditis and revealed that 8% of individuals had pathogenic/likely pathogenic (P/LP) variants in one of the sarcomere genes, and approximately half of them were *TTN*tv/lof variants (4.17%) [8]. Due to the enormous size of *TTN*, rare and unique variants occur with a high frequency and approximately 1% of the population harbor *TTN*tv [9]. However, in patients with myocarditis, these variants are detected 10 times more often, underlining the role of molecular structural defects of cardiomyocytes in the development of myocarditis.

Titin is specifically degraded by proteases such as trypsin and calpain [10,11]. As a consequence, circulation titin fragments reflect the damage to myocytes in contractile tissues. It has been known for over a decade that titin serum concentration is an informative marker of disease severity and progression in several neuromuscular disorders, such as Duchenne and Becker muscular dystrophies, spinal muscular atrophy and others. In addition, titin serum concentration was proposed as a predictive factor for sarcopenia and exercise-induced muscle damage [12,13]. To assess the breakdown of myocytes/cardiomyocytes in the above-mentioned diseases, as well as in other pathological conditions, such as amyotrophic lateral sclerosis, interstitial lung diseases or after cardiac surgery, the ratio of serum titin to creatinine is typically used to account for renal filtration capacity [14,15,16]. There are only a few reports specifying serum titin as a marker of myocardial damage and prognosis, for example, in acute myocardial infarction or in diabetes-associated cardiac damage [17,18]. Here we hypothesize that titin serum level increases in patients with myocarditis and question whether this can depend on the potential genetic background of the disease. To test this hypothesis, we performed genotyping in patients with myocarditis using targeted next-generation sequencing and analyzed their circulating titin level in relation to genetic etiology, degree of myocardial dysfunction and recovery.

## 2. Materials and Methods

### 2.1. Participants

A total of 46 patients with myocarditis were included in this study. The diagnosis was defined based on the position statement of the ESC Working Group on Myocardial and Pericardial Diseases (2013) [19]. The inclusion criteria were: age over 18 years, dilated cardiac phenotype, left ventricular ejection fraction less than 40%, absence of family history of cardiomyopathy and morphological confirmation of lymphocytic myocarditis by endomyocardial biopsy [19]. The control group for immuno-enzyme analysis consisted of 12 age-matched healthy donors. Clinical data were collected from electronic health records.

### 2.2. Genetic Testing

For genetic testing, the DNA was extracted from whole blood with a FlexiGene Kit (QuiGen, Venlo, The Netherlands) according to the manufacturer’s recommendations. A targeted panel of 172 cardiomyopathy-associated genes was analyzed using the SureSelect Target Enrichment System (Agilent; Waldbronn, Germany) with an Illumina MiSeq instrument [20]. The list of studied genes is presented in Supplemental Table S1. The pathogenicity of the variants was interpreted according to American College of Medical Genetics and Genomics and the Association for Molecular Pathology guidelines [21]. Pathogenic (P), likely pathogenic (LP) variants and variants of uncertain significance (VUSs) in genes linked to cardiomyopathy phenotype were considered for further analysis.

### 2.3. Serum Samples and Measurement of Serum Titin and Creatinine Levels

Serum titin levels were measured in a subgroup of 27 patients in which serum samples were available for the analysis. For each patient, one serum titin value was used for analysis. Serum samples were obtained in parallel with routine clinical blood tests and stored at −20 °C until analysis. Serum titin levels were measured using the Human Titin Enzyme-Linked Immunosorbent Assay (ELISA) kit (QT-EH1877) (FineTest, Wuhan, China), according to the manufacturer’s instructions.

Serum creatinine (Cr) levels were measured using an biochemical automatic analyzer, the BS-200E (Mindray, Shenzhen, China), using original Mindray reagents. The serum titin/Cr ratio was calculated by dividing the serum titin concentration by the serum Cr concentration obtained in parallel and expressed in ng/mkmol Cr.

### 2.4. Morphological Examination and Viral Genome Detection

The endomyocardial biopsy was performed in accordance with the protocol described in our previous publication [22]. The determination of viruses and CD3 lymphocytes was carried out by immunohistochemical analysis using appropriate antibodies: CD3 (rabbit polyclonal antibody, DAKO, Nottingham, UK; dilution 1:100), enteroviral VP1 (mouse monoclonal antibody, 5-D8/1; DAKO; UK dilution 1:25), anti-B19 (rabbit polyclonal antibody, DAKO, Nottingham, UK; dilution 1:50), and HHV6 antibody (Mouse monoclonal antibody, C3108-103, ABCAM, CA, USA; dilution 1:25).

### 2.5. Statistics

Statistical analysis was performed using Stata 18 software (StataCorp LLC, College Station, TX, USA). The normality of data distribution was assessed using the Shapiro–Wilk test alongside visual inspection of histograms. Categorical variables are presented as frequencies and percentages (*n*, %). Continuous variables are expressed as either means ± standard deviations (SDs) or as medians with quartiles (25th–75th percentiles).

Categorical variables were compared using Fisher’s exact test. For multi-group comparisons of continuous variables, the Kruskal–Wallis test was applied. Post hoc pairwise intergroup comparisons were performed using the Mann–Whitney U test with a Bonferroni correction. Differences were considered statistically significant at *p* < 0.05. Relationships between continuous variables were evaluated using Spearman’s rank correlation. Time-to-event analysis was conducted using the Kaplan–Meier method with the log-rank test. The impact of continuous variables on time-to-event outcomes with censored data was assessed using univariable Cox proportional hazards regression. ROC curve analysis was performed to assess the diagnostic performance of the indicator. The value for testing the null hypothesis was determined using the asymptotic z-test. The optimal threshold value was determined using the Youden index.

## 3. Results

### 3.1. Baseline Characteristics of the Group

The study included 46 patients with morphologically proved myocarditis confirmed using endomyocardial biopsy, of whom 33 were male (71.7%). Clinical characteristics of the group are presented in Table 1. The average age at the time of diagnosis was 41.8 ± 12.2 years. Most of the patients reported an association between disease onset and acute infection episode; chest pain at presentation was reported in 33% of patients. At the time of admission, the left ventricular ejection fraction was 26.5 ± 9.6%; detailed echo-cardiography (EchoCG) and magnetic resonance imaging (MRI) parameters are presented in Supplemental Table S2. Atrial fibrillation developed in 28% of the patients; ventricular arrhythmias were observed in 74%, including episodes of sustained ventricular tachycardia registered in 24% of the cases. Myocardial biopsy showed necrosis in 26% of the samples, cardiomyocyte hypertrophy in 76% and fibrosis in 24%. Immunohistochemical analysis revealed the presence of the viral genome in 65% of biopsy samples, including Epstein–Barr virus, parvovirus and human herpesvirus 6. A substantial proportion of the patients received immunosuppressive therapy and none was treated with antiviral drugs. To assess the disease outcomes, the endpoints were death and heart transplantation (performed or inclusion in the list). Over time, 20% of the patients reached one of the endpoints—6 (13%) deaths and 3 (7%) underwent Htx.

### 3.2. Genetic Analysis of Patients with Myocarditis

The genotyping performed in all 46 patients revealed the presence of pathogenic variants, likely pathogenic variants and variants of the uncertain significance in 31 out of 46 patients (67%, Figure 1). Overall, 47 variants were identified, of which 1 was pathogenic (P), 12 were likely pathogenic (LP) and 34 were assessed as variants of uncertain significance (VUSs). Of these 47 variants, 6 were detected in the *TTN* gene, 5 of which were likely pathogenic variants with loss-of-function mechanism of action (Figure 1). Thus, in our cohort, the frequency of *TTN*tv/lof in patients with myocarditis corresponded to 11%. All detected *TTN*tv/lofs were located in exones, 100 percent of which were spliced in parameter. In addition to *TTN*tv/lof, P and LP variants were detected in cytoskeletal and structural genes such as *FLNC*, *DSP*, *ANK2*, *NEBL*, *AKAP9* and *SYNE1.* Notably, all of them were variants with loss-of-function mechanism predicting either splicing defects or stop codon gain. In total, 13 loss-of-function P/LP variants, predominantly in structural or cytoskeletal genes (except for *GAA* and *PKD1L1*), were detected in patients with myocarditis, corresponding to 28%. Importantly, no P/LP variants were detected either in contractile sarcomeric genes such as *MYH7*, *MYBPC3* and troponin complex genes, or in *LMNA* gene.

To analyze possible genotype–phenotype associations, all patients were divided into three groups according to their genotype: patients harboring *TTN*tv*/*lof variants (*TTN*tv/lof, *n* = 5), genotype-positive patients excluding *TTN*tv/lof variants (*n* = 26) and genotype-negative cases (*n* = 15). If a patient had a combination of several variants, the patient was classified based on the carriage of *TTN* tv/lof. Clinical characteristics of the groups are presented in Table 2.

We detected a significant difference between *TTN*tv/lof group and genotype-negative group in the change in end-diastolic volume (∆EDV) after 6 months of treatment (Mann–Whitney U test, Bonferroni-adjusted *p* = 0.038) (Figure 1b). The frequency of morphologically confirmed myocardial necrosis differed significantly between genotype-negative patients and carriers of non-TTNtv/lof variants (Fisher’s exact test, Bonferroni-adjusted *p* = 0.045) (Figure 1c).

There was no significant difference in survival between the genotype-positive and genotype-negative groups based on the log-rank test for the composite endpoint of heart transplantation or cardiovascular death (χ^2^ = 0.6, *p* = 0.44).

### 3.3. Serum Titin Level in Patients with Myocarditis

The analysis of circulating serum titin concentration was performed in a subgroup of 27 patients with myocarditis, in which the serum was collected and available for analysis, and in 12 healthy donors who served as a control group. Both serum titin concentration and the titin/creatinine ratio were used for subsequent analysis. A significant increase in both titin and titin/creatinine was observed in the group of patients with myocarditis compared with the control group (Table 3, Figure 2a,b).

ROC analysis was performed to evaluate the ability of titin level to discriminate between patients with myocarditis and healthy controls. The area under the ROC curve (AUC) for titin was 0.824, which was slightly higher than the AUC of 0.799 observed for the titin/creatinine ratio, with *p* value < 0.001 for both (Figure 2b,c). The optimal titin cutoff value, determined by the highest Youden’s index, was ≥69.489 ng/mL, yielding a sensitivity of 81.5% and a specificity of 83.3% (Youden’s index = 0.648). Additionally, a highly specific titin threshold of ≥91.896 ng/mL was identified; it demonstrated 100% specificity, though sensitivity decreased to 55.6%. Consequently, titin values ≥91.896 ng/mL can be used primarily to rule in (confirm) myocarditis, whereas lower values do not allow ruling out the disease. The optimal titin/creatinine ratio cutoff value, determined by the maximum Youden’s index, was ≥0.8328 ng/mkmol. At this threshold, the sensitivity was 70.4%, specificity was 91.7%, and Youden’s index was 0.620.

Patients who achieved full recovery (LV EF by Simpson > 50% after six months) demonstrated significantly lower levels of serum titin compared to the patients without full recovery (Mann–Whitney, *p* = 0.046). Two patients who died from cardiovascular causes or underwent heart transplantation prior to the 6-month follow-up were automatically classified into the non-recovery group. We also detected a difference in serum titin level in patient groups depending on clinical parameters; thus, patients without ventricular arrhythmias and patients with full recovery demonstrated significantly lower levels of serum titin compared to patients presenting with ventricular arrhythmias and non-recovered patients (Figure 3a,b).

We also performed univariable Cox proportional hazards regression analysis to evaluate the association between TTN level and the composite endpoint, defined as death or heart transplantation. Median follow-up duration was 62 months (95% CI, 45–73 months). During follow-up, the median event-free survival was not reached and the composite endpoint occurred in six patients (22.2%) (two patients underwent heart transplantation and four patients died). In univariable Cox proportional hazards regression analysis, TTN level was not significantly associated with the risk of the composite endpoint (HR = 1.004, 95% CI 0.994–1.014, likelihood-ratio χ^2^ = 0.54, *p* = 0.462).

In addition, we revealed the correlations of titin and titin/creatinine ratio with left ventricular ejection fraction and left ventricular reverse remodeling, defined as the change in ejection fraction after treatment by Spearman’s correlation (Figure 3c–f).

When levels of titin were compared among the different genotype groups, a difference was found between genotype-positive patients without *TTN*tv/lof and the control group (Mann–Whitney U test, adjusted *p* = 0.045) and between genotype-negative patients and the control group (Mann–Whitney U test, adjusted *p* = 0.011) (Table 4, Figure 4). Notably, there was no difference between the control group and patients with *TTNtv* (Mann–Whitney U test, adjusted *p* = 0.45) (Table 4, Figure 4). A difference in serum titin level was also observed between genotype-negative and all genotype-positive patients (adjusted *p* = 0.040), or between genotype-positive patients and the control group (adjusted *p* = 0.0204) (Table 4). Comparison of titin/creatinine ratio between genetic subgroups revealed the difference only between genotype-negative and control subjects (adjusted *p* = 0.011) (Table 4, Figure 4).

To summarize, we detected that serum titin level is elevated in patients with myocarditis compared to controls, and is associated with the development of ventricular arrhythmias, a low rate of myocardial reverse remodeling being the highest in the group of genotype-negative patients and corresponding to almost normal rates in patients harboring *TTN*tv/lof variants.

## 4. Discussion

The contemporary concept of myocarditis combines the role of genetic background and autoimmune-mediated inflammation [1,3,8]. Neither of these components alone can explain the origin or triggering factors of myocarditis, its persistence, progression or resolution. The understanding of myocardial inflammation and healing based only on infectious or autoimmune hypothesis and antiviral or immunosuppressive treatment did not result in sufficient improvement of patient outcomes and does not currently make it possible to stratify patients according to their prognosis. At the same time, the view of myocarditis solely as aseptic inflammation in the light of genetically determined cardiomyopathy does not make it possible to abolish the inflammatory progression early in time and to prevent maladaptive myocardial remodeling upon concurrent viral and bacterial infections. The urgent cotemporary need for a more detailed deciphering of the molecular pathogenesis of myocarditis in the era of high-throughput genetic analysis and multiomics technologies is based on the great clinical demand for proper patient guidance for innovative treatment strategies, prospective follow-up, clinical genetic counseling and timely planning of surgical interventions [23]. To address this need, one should utilize more informative biomarkers of myocarditis etiology and progression. The list of such biomarkers with regard to myocardial inflammation is currently quite limited and nonspecific and includes widely accepted inflammatory markers and cell injury markers, such as C-reactive protein and troponin I. For more thorough diagnostics, endomyocardial biopsy is accepted as a gold standard, especially in combination with MRI. However, this combination of costly, invasive and operator-dependent technologies makes its availability restricted only to highly specialized medical centers. As a consequence, the finding of the new molecular markers, potentially informative to delineate the pathogenesis of myocarditis and to weigh the impact of genetic and autoimmune factors, will not only guide patient treatment but also help uncover the nature of this still largely unknown disorder of myocardial tissue.

There is sufficient evidence now that cardiomyopathy-associated mutations constitute the genetic basis of myocarditis [1,8,24,25,26]. The proportion of genotype-positive cases with variants in cardiomyopathy-associated genes in biopsy-proven myocarditis varies depending on the study cohort, age of presentation and genotyping approach ranging from 8% to 31%, with loss-of-function variants predominating [2,8]. In this regard, our data, showing a proportion of P/LP variants in patients with myocarditis as 28%, are consistent with the majority of previously published reports. However, several significant differences were detected in regard to clinical presentations, prognosis and genes identified. Thus, Kontorovich et al. described no difference in disease severity and outcome between genotype-positive and genotype-negative patients [25], while Seidel and co-authors reported a worse prognosis for patients with myocarditis associated with P/LP variants in cardiomyopathy-associated genes. In contrast, in our study, the genotype-positive group of patients was characterized by a more benign clinical course. One of the possible explanations can be the inclusion of pediatric cohorts in the first two studies, when the genetic etiology of the disease and possible genotype–phenotype associations are different in pediatric and adult groups. This hypothesis is further supported by the fact that variants in contractile sarcomeric genes such as *MYH7*, *TNNI3* and *TNNT2* were detected only in the pediatric cohort with myocarditis, in contrast to adult groups characterized mainly by LOF variants detected in *TTN*, *DSP* and other cytoskeletal and structural genes [1,8,25,26]. Together with previously published cohorts, our data support the role of LOF variants in cardiomyopathy-associated genes in the predisposition to myocardial inflammation [27]. The frequency of *TTN*tv/lof in the myocarditis group was reported as 6% by Kantorovich and co-authors, while in our cohort, it was almost twice as high and corresponded to 11%. Importantly, *TTN*tv/lofs are detected in approximately 1% of the population, and our previous studies in an ethnically matched cohort defined the *TTN*tv/lof population rate as 0.52% [9]. Thus, similar to other reports focused on *TTN*tv/lof variants in patients with cardiac disorders, in our cohort, the rate of *TTN*tv/lof in patients with myocarditis was at least 20 times higher than the population rate. Notably, the proportion of viral genome-associated cases in genotype-positive and genotype-negative patients was similar in our cohort and in previously published studies [24], suggesting the concomitant, but not primary, causative role of viral infection in pathogenesis of myocarditis.

Importantly, we found a significant difference in clinical course and prognosis between *TTN*tv/lof and genotype-negative cases when patients with *TTN*tv/lof had better myocardial reverse remodeling and less myocardial necrosis according to endomyocardial biopsy. In general, it supports the hypothesis of a better prognosis of *TTN*tv/lof-associated cardiac dilated phenotype with a good response to optimal heart failure treatment [28]. In our cohort, the genotype-negative status of myocarditis was associated with more aggressive immune inflammation reflected in a greater burden of morphologically proven necrosis. Therefore, we propose that these genotype-negative cases represent the “true” immune myocarditis with more aggressive inflammation. To extrapolate this hypothesis, we further focused on the search of biochemical markers letting to reliably identify this group.

We demonstrated that titin serum level is elevated in patients with myocardial injury and the degree of this elevation is associated with the patient’s genotype: carriers of *TTN*tv/lof had almost normal titin serum level compared to other genotype-positive cases, where it was significantly elevated compared to controls and to *TTN*tv/lof carriers (Table 4, Figure 2). The highest titin serum level was detected in genotype-negative cases who had a greater burden of necrosis. This observation can have several potential explanations. First, patients with the *TTN*tv/lof genotype may have lower myocardial titin content due to a haploinsufficiency mechanism and, consequently, lower titin serum concentration due to cardiomyocyte damage. Second, patients with *TTN*tv/lof may have a lower level of cardiomyocyte damage and cell lysis compared to patients with more aggressive cellular infiltration and immune-mediated damage, leading to less release of cardiac markers into the circulation. The comparison of titin serum level with ultrasensitive troponin can potentially help in addressing this question. Nevertheless, serum TTN concentration may reflect the degree of cell injury and lysis, representing an additional cardiomyocyte injury marker, such as troponin, myoglobin and creatine kinase-MB. Notably, the titin/Cr ratio was reported to be more informative in several neuromuscular disorders, and acute cardiac injury did not outperform TTN level in discriminating patients with myocarditis from healthy controls in our cohort. However, larger clinical groups and more thorough comparison with other serum markers, such as ultrasensitive troponin, myoglobin, creatinekinase-MB, are needed to validate the observed association.

One of the important associations detected was the inverse correlation of titin serum level with contractility improvement (∆ left ventricular ejection fraction). Thus, patients with higher titin level were less likely to recover contractility after six months and were more likely to have clinically significant reverse remodeling. Accordingly, patients with low serum titin level have a higher chance to reduce myocardial dilation in response to heart failure treatment. Nevertheless, in our univariable Cox proportional hazards regression analysis, titin level did not predict long-term event-rate, which was driven to a greater extent by cardiovascular death than by heart transplantation. This may be explained by the low power of the study or could indicate that titin level better predicts only early or mid-term myocardial reverse remodeling rather than long-term survival, where not only heart failure, but also competing risk of sudden cardiac death, contributes. This hypothesis could be validated in future, more powerful studies. Together with the observed thresholds for sensitivity and specificity of titin serum concentrations according to ROC analysis, the detection of circulating titin may be an additional promising biomarker not only for detecting myocarditis but also for predicting patient prognosis and treatment effect. In case of further validation in larger clinical groups and different myocardial pathologies, titin serum level can have a diagnostic marker potential in addition to troponin of NT-proBNP to stratify patients with myocarditis.

## 5. Conclusions

In summary, in our cohort of patients with biopsy-proven myocarditis, the rate of LOF variants was 28% with a clear predominance of variants in structural and cytoskeletal genes and *TTN*tv/lofvariants being detected in 11% of patients. The latter group was characterized morphologically by less necrosis and inflammation and more favorable myocardial reverse remodeling. Additionally, we demonstrated that serum titin level may have potential as a biomarker for distinguishing patients with myocarditis and its high level is associated with worse clinical course: less myocardial reverse remodeling and higher prevalence of ventricular arrhythmias. The genotype-negative status of patients was characterized by the highest serum titin level and titin/Cr ratio, less degree of myocardial reverse remodeling, sustained ventricular arrhythmias, marked inflammatory cell infiltration and higher area of necrosis.

## 6. Limitations

Unfortunately, our study has a number of limitations; in particular, we did not measure the level of anti-myocardial antibodies in the patients, and we also did not bank urine samples for studies that could be useful in analyzing this group, such as measuring the N-terminal fragment of titin. Serum levels of ultrasensitive troponin, myoglobin, and creatine kinase also were not analyzed. One of the key limitations is the small size of the groups according to their genotypes, especially the *TTN*tv/lof group (five patients in total and only four eligible for serum TTN analysis). However, considering the prevalence of *TTN*tv/lof-associated cardiac dysfunction among patients with heart failure, we consider these pilot data from a small number of patients to be of high clinical importance and worthy of reporting. In future studies, we plan to take into account all the aforementioned limitations, conduct more detailed laboratory studies in the larger patient groups and compare patients with lymphocytic myocarditis with those suffering from other cardiological diseases.

## Figures and Tables

**Figure 1:**
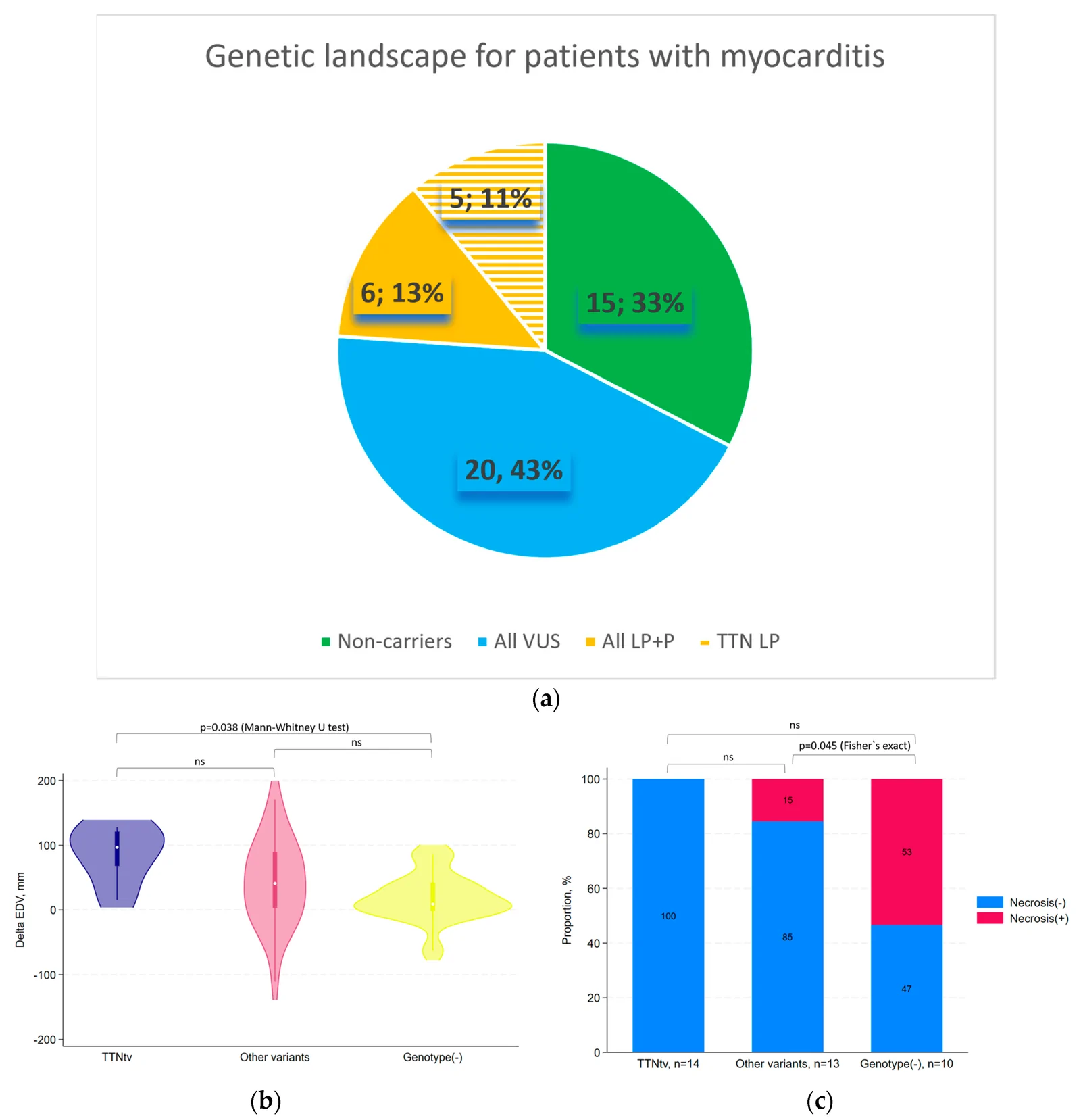
Genetic spectrum of patients with myocarditis and genotype–phenotype correlations according to genetic testing results. (**a**) Prevalence of pathogenic (P) variants, likely pathogenic (LP) variants and variants of unknown significance (VUSs) in patients with myocarditis. (**b**) Comparison of myocardial reverse remodeling, reflected by the change in end-diastolic volume (ΔEDV), according to genotype by pairwise Mann–Whitney U test. ΔEDV was calculated as follows: EDV initially—EDV after 6 months of therapy. (**c**) Comparison of morphologically confirmed myocardial necrosis frequency according to genotype by pairwise Fisher’s exact test. ns—not significant. Presented *p* values are Bonferroni-adjusted.

**Figure 2:**
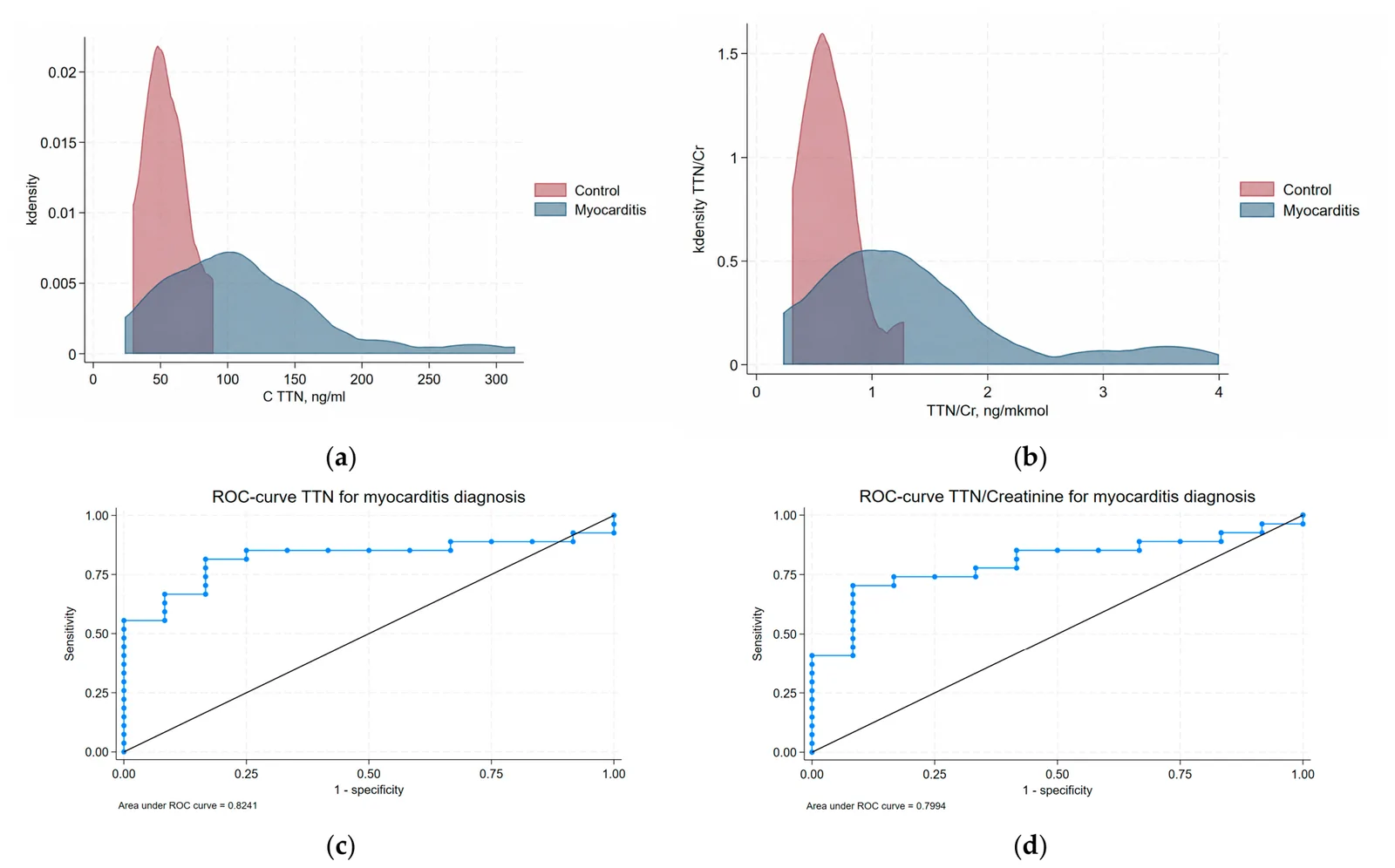
Titin level and titin/Cr ratio and receiver operating characteristic (ROC) analysis in patients with myocarditis. Kernel density plots for titin levels (**a**) and the titin/creatinine (**b**) ratio in myocarditis (*n* = 27) and control groups (*n* = 12). (**c**) ROC curve demonstrating the potential of titin serum level to distinguish patients with myocarditis from healthy donors. (**d**) ROC curve demonstrating the potential of titin/Cr ratio to distinguish patients with myocarditis from healthy donors. The blue line shows the receiver operating characteristic (ROC) curve, and the black diagonal line indicates the reference line corresponding to no discriminatory ability.

**Figure 3:**
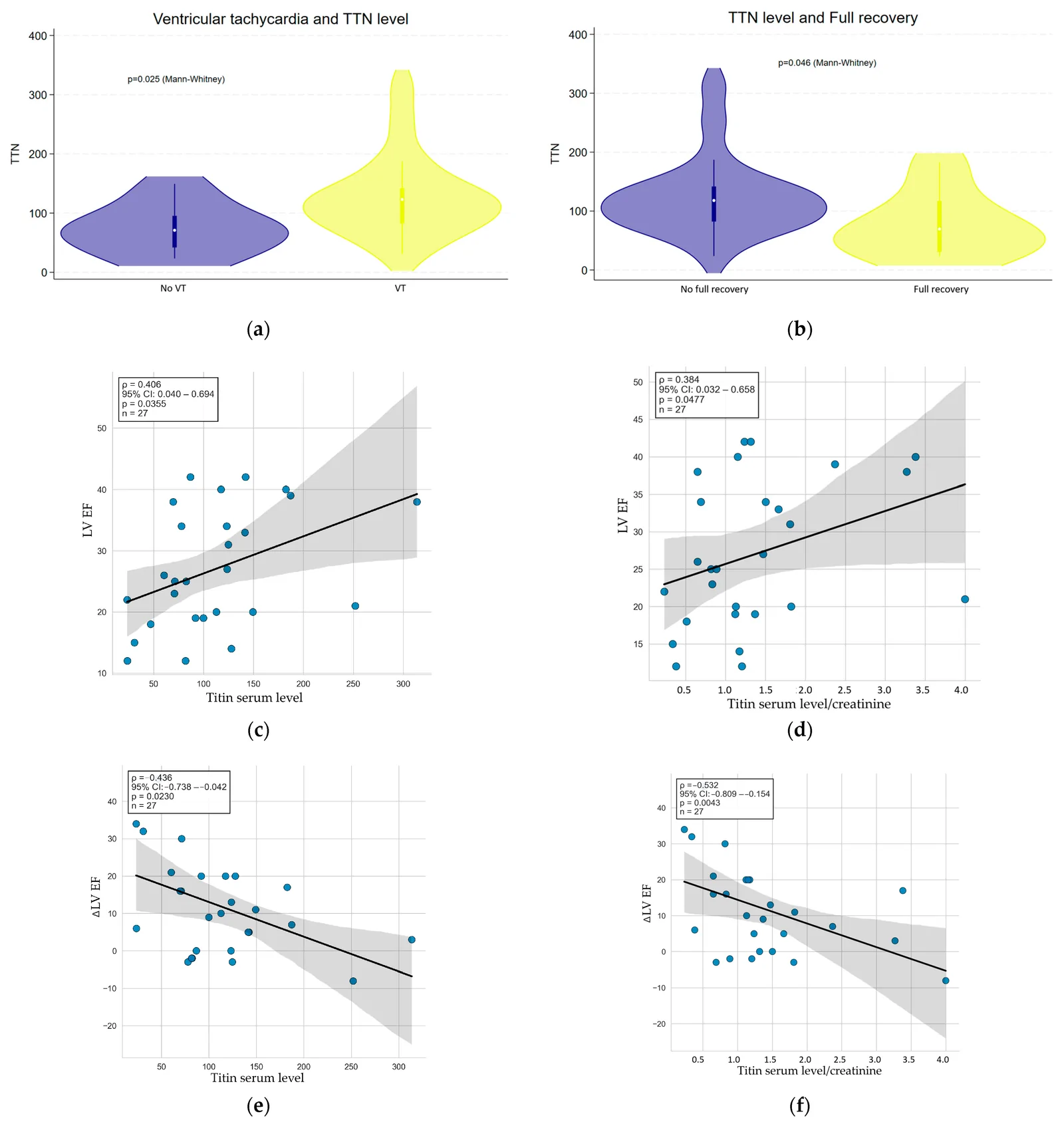
Correlations of echocardiographic parameters with titin serum level. (**a**) Titin serum level in patients depending on the presence of ventricular arrhythmias. (**b**) Titin serum level in patients with and without full recovery (achievement of LV EF >50% after 6 months). (**c**) Correlation of left ventricular ejection fraction with serum titin level. (**d**) Correlation of left ventricular ejection fraction with serum titin/creatinine ratio. (**e**) Correlation of change in left ventricular ejection fraction with serum titin level. (**f**) Correlation of changes in left ventricular ejection fraction with the serum titin/creatinine ratio. Blue circles indicate individual observations; the black line shows the fitted regression line, and the gray shaded area indicates the 95% confidence interval.

**Figure 4:**
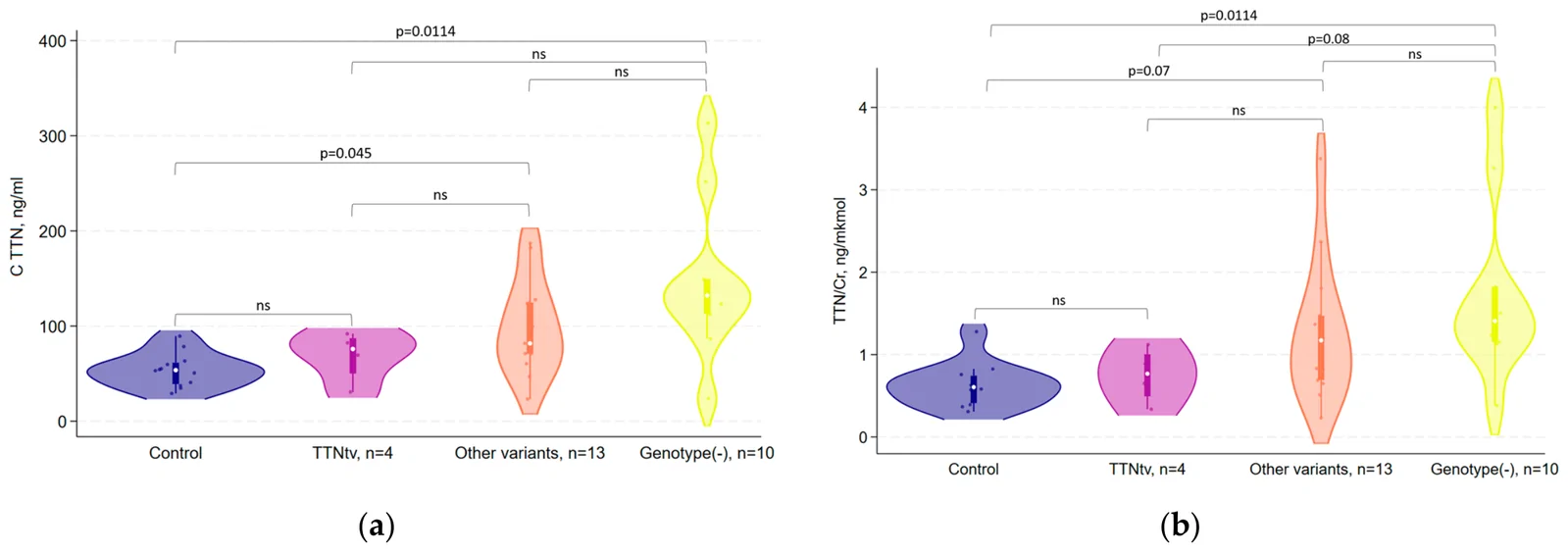
Comparison between genetic subgroups of patients with myocarditis and the control group. (**a**) By serum titin level. (**b**) By serum titin/creatinine ratio. ns—not significant.

**Table 1:** Clinical characteristics of the group.

	*n* = 46
Age at presentation, years	41.8 ± 12.2
Sex, male, *n* (%)	33 (72)
Link with infection, *n* (%)	33 (72)
Heart failure III/IV (NYHA), *n* (%)	38 (83)
Chest pain, *n* (%)	15 (33)
Syncope, *n* (%)	16 (35)
Abnormal TrI at presentation, *n* (%)	8 (17)
Systemic hypotension or cardiogenic shock, *n* (%)	12 (26)
Ventricular arrhythmias, *n* (%)	34 (74)
Sustained ventricular tachycardia, *n* (%)	11 (24)
Atrial fibrillation, *n* (%)	13 (28)
EF LV ^1^ at presentation, %	25 [19; 34]
End-diastolic volume, mL	230 [181; 268]
Endomyocardial biopsy	
Cardiomyocyte necrosis, *n* (%)	12 (26)
Fibrosis, *n* (%)	39 (85)
CD3-lymphocytes, cell/m^2^	19 [14; 26]
Viral genome, *n* (%)	30 (65)
Steroid therapy, *n* (%)	18 (39)
Immunoglobulin i/v, *n* (%)	11 (24)
Cytostatic therapy, *n* (%)	15 (33)
NT-proBNP ^2^, pg/mL	2259 [981; 5286]
Full recovery	12 (26)
Death, *n* (%)	7 (15)
Heart transplantation, *n* (%)	3 (7)

^1^ EF LV—left ventricular ejection fraction, ^2^ NT-proBNP—N-terminal prohormone of brain natriuretic peptide.

**Table 2:** Clinical significance of myocarditis genetic subgroups.

	All Genotype- Positive (*n* = 31)	Genotype- Negative (*n* = 15)	TTNtv/lof(+) (*n* = 5)	Other Variants (*n* = 26)	P Omnibus (Group 2vs3vs4)
No of Group	1	2	3	4	
Age, years	42 [33; 49]	43 [32; 59]	37 [36; 41]	42 [32; 49]	ns
Sex, male (%)	21 (68)	11 (73)	4 (80)	17 (65)	ns
Link with infection, *n* (%)	23 (74)	10 (67)	5 (100)	18 (69)	ns
Heart failure III/IV (NYHA), *n* (%)	25 (81)	13 (87)	4 (80)	21 (81)	ns
Spec. therapy (GCS ^1^, IVIG),%	11 (35), 5 (16)	7 (47), 6 (40)	2 (40), 2 (40)	9 (35), 3 (12)	ns
Death/heart transplantation, %	5 (16)	4 (27)	1 (20)	4 (15)	
ECG ^2^, EchoCG ^3^					
Low voltage, *n* (%)	3 (10)	1 (7)	2 (40)	1 (4)	0.075
Atrial fibrillation, *n* (%)	9 (29)	4 (27)	2 (40)	7 (27)	ns
Unsustained ventricular arrhythmias, *n* (%)	6 (19)	5 (33)	0 (0)	6 (23)	ns
Unsustained ventricular arrhythmias, *n* (%)	12 (39)	4 (27)	4 (80)	8 (31)	ns
EF LV ^4^ initially, %	26 [19; 34]	23 [20; 36]	19 [18; 25]	27 [19; 34]	ns
EF LV after therapy,	44 [30; 49]	34 [24; 42]	47 [39; 54]	44 [29; 48]	ns
%∆ EF LV, %	13 [5; 21]	5 [0; 12]	20 [16; 32]	11 [5; 20]	ns
EDV ^5^ initially, ml	236 [202; 277]	197 [178; 249]	268 [246; 289]	231 [201; 264]	ns
EDV after therapy, ml	180 [140; 232]	180 [151; 203]	171 [155; 182]	190 [140; 239]	ns
∆ EDV, mL	56 [5; 91]	9 [−2; 42] *	97 [68; 121] *	41 [3; 90]	0.046
Endomyocardial biopsy					
Cardiomyocyte necrosis, *n* (%)	4 (13)	8 (53) ^†^	0 (0)	4 (15) ^†^	0.013
CD3-lymphocytes, cell/mm^2^	18 [14,24]	19 [15,38]	15 [13,20]	18 [14,24]	ns
CD3-lymphocytes > 30 cell/mm^2^, *n* (%)	5 (16)	6 (40)	0 (0)	5 (19)	ns
Laboratory tests					
Virus genome, *n* %	20 (65)	10 (67)	3 (60)	17 (65)	ns
Enterovirus, *n* (%)	16 (51)	10 (67)	3 (60)	13 (50)	ns
Parvovirus, *n* (%)	10 (32)	8 (53)	2 (40)	8 (31)	ns
Herpesvirus, *n* (%)	8 (26)	3 (20)	1 (20)	7 (27)	ns
NT-proBNP ^6^, pg/mL	1232 [696; 5286]	2433 [1152; 3018]	3280 [1112; 5180]	1210 [696; 5560]	ns

^1^ GCS—glucocorticosteroids, ^2^ ECG—electrocardiography, ^3^ EchoCG—echocardiography, ^4^ EF LV—left ventricular ejection fraction, ^5^ EDV—end-diastolic volume, ^6^ NT-proBNP—N-terminal prohormone of brain natriuretic peptide, *—*p* < 0.05 between genotype-negative and *TTN*tv/lof(+), ^†^—*p* < 0.05 between genotype-negative patients and patients with non-TTNtv/lof variants (other variants). All *p* values in post hoc analysis are Bonferroni-adjusted. Correction for multiple testing of hypothesis was not performed. ∆EDV was calculated as follows: EDV initially–EDV after 6 months of therapy. ns—not significant.

**Table 3:** Comparison of the titin level and the titin/creatinine ratio between patients with myocarditis and the control group.

	Myocarditis (*n* = 27)	Control (*n* = 12)	*p* Value
C (Titin), ng/mL	99.8 [70.8; 141.4]	53.7 [39.1; 61.6]	0.001
Titin/Cr, ng/mkmol	1.2 [0.7; 1.7]	0.6 [0.4; 0.7]	0.003
Age, years	42 [32; 53]	39 [35; 46]	0.75
Male sex, *n* (%)	18 (67)	9 (75)	1.0

The data are presented as medians [q1; q3] because of their nonparametric distribution.

**Table 4:** Comparison of the titin level and the titin/creatinine ratio between subgroups of patients with myocarditis and the control group.

	Control (*n* = 12)	TTNtv/lof+ (*n* = 4)	All Genotype- Positive (*n* = 17)	Genotype-Positive Without TTNtv/lof+ (*n* = 13)	Genotype- Negative (*n* = 10)
*TTN*, ng/mL	53.7 [39.1–61.6] ^^†×^	76.0 [50.1–87.2]	81.8 [69.5–123.6] ^×o^	81.8 [70.8–124.7] ^†^	132.2 [112.7–149.3] ^^o^
Cr,mkmol/L	87.9 [83.8–96.3]	93.3 [88.8–96.5]	87.1 [79–93]	85.2 [73–93]	85.3 [70–99]
*TTN*/Cr, ng/mkmol	0.6 [0.4–0.7] ^^×^	0.8 [0.5–1.0]	0.9 [0.6–1.4] ^×^	1.2 [0.7–1.5]	1.4 [1.2–1.8] ^

^×^—*p* < 0.05 between all genotype-positive patients and control group; ^†^—*p* < 0.05 between patients without *TTN*tv/lof and control group; ^—*p* < 0.05 between genotype-negative patients and control group; ^o^—*p* < 0.05 between genotype-negative and all genotype-positive patients. All *p* values in post hoc analysis are adjusted for multiple testing.

## Data Availability

The datasets presented in this article are not readily available because it is not allowed according to a national law. Requests to access the datasets should be directed to The Federal Medical–Biological Agency of Russia.

## Supplementary Materials

The following supporting information can be downloaded at: https://www.mdpi.com/article/10.3390/jcdd13100487/s1, Table S1: List of studied genes; Table S2: EchoCG and MRI results.

## Abbreviations

The following abbreviations are used in this manuscript:

*TTN*tv/lof	Titin gene truncating or loss-of-function variant
LOF	Loss of function
ELISA	Enzyme-Linked Immunosorbent Assay
Cr	Serum creatinine
AF	Atrial fibrillation
EchoCG	Echocardiography
MRI	Magnetic resonance imaging
EDV	End-diastolic volume
AUC	Area under the ROC curve
P	Pathogenic
LP	Likely pathogenic
VUSs	Variants of uncertain significance
LV EF	Left ventricular ejection fraction
