# Supplementary material for: Titin Serum Level Is Elevated in Patients with Myocarditis Depending on Their Genetic Background
Source: J Cardiovasc Dev Dis. 2026 Sep 28;13(10):487. doi: 10.3390/jcdd13100487 (PMC13705468; doi:10.3390/jcdd13100487)
Supplement: Supplementary file 1 [file jcdd-13-00487-s001.zip › Supplemental Table S1 List of studied genes.pdf]

Supplementary Table S1
List of studied genes.

| | Gene name | Interval | Regions | Size |
|---|---|---|---|---|
| 1. | ABCC9 | chr12:21801034-21936684 | 41 | 5759 |
| 2. | ACADVL | chr17:7217136-7225107 | 20 | 2735 |
| 3. | ACTA1 | chr1:229431489-229433125 | 6 | 1254 |
| 4. | ACTC1 | chr15:34790402-34794818 | 6 | 1254 |
| 5. | ACTN2 | chr1:236686664-236762629 | 23 | 3342 |
| 6. | ACVR2B | chr3:38454313-38483342 | 11 | 1759 |
| 7. | AGK | chr7:141555457-141652934 | 17 | 1670 |
| 8. | AKAP9 | chr7:91941090-92110169 | 50 | 13065 |
| 9. | ALPK3 | chr15:84816837-84868466 | 14 | 6004 |
| 10. | ANK2 | chr4:112904484-113381650 | 56 | 13912 |
| 11. | ANKRD1 | chr10:90912856-90921037 | 9 | 1140 |
| 12. | ANO5 | chr11:22193483-22279775 | 22 | 3182 |
| 13. | BAG3 | chr10:119651666-119677292 | 5 | 1834 |
| 14. | BRAF | chr7:140726484-140924713 | 21 | 2799 |
| 15. | CACNA1C | chr12:1971053-2691209 | 56 | 8643 |
| 16. | CACNA2D1 | chr7:81950382-82443469 | 41 | 4204 |
| 17. | CACNB2 | chr10:18140727-18539734 | 20 | 2669 |
| 18. | CALM1 | chr14:90397221-90404727 | 7 | 596 |
| 19. | CALM2 | chr2:47160766-47176551 | 8 | 929 |
| 20. | CALM3 | chr19:46601425-46609163 | 7 | 593 |
| 21. | CALR3 | chr19:16479121-16496139 | 9 | 1335 |
| 22. | CASQ2 | chr1:115701231-115768551 | 11 | 1420 |
| 23. | CAV3 | chr3:8733867-8745877 | 2 | 496 |
| 24. | CBL | chr11:119206408-119308087 | 18 | 3163 |
| 25. | CDH2 | chr18:27952143-28177032 | 18 | 3179 |
| 26. | CMYA5 | chr5:79689898-79799626 | 13 | 12470 |
| 27. | CRELD1 | chr3:9934429-9944589 | 11 | 1697 |

| | | | | |
|---|---|---|---|---|
| 28. | CRYAB | chr11:111908754-111911734 | 4 | 867 |
| 29. | CSRP3 | chr11:19182660-19192458 | 5 | 685 |
| 30. | CTNNA3 | chr10:65920320-67648780 | 19 | 3212 |
| 31. | DES | chr2:219418453-219426000 | 9 | 1593 |
| 32. | DMD | chrX:31121823-33339275 | 87 | 13178 |
| 33. | DMPK | chr19:45770471-45782381 | 16 | 2715 |
| 34. | DNAAF1 | chr16:84145431-84178416 | 15 | 2639 |
| 35. | DNAAF3 | chr19:55159052-55166658 | 12 | 2146 |
| 36. | DPP6 | chr7:153887674-154892490 | 29 | 3721 |
| 37. | DSC2 | chr18:31068005-31101981 | 17 | 3082 |
| 38. | DSG2 | chr18:31498242-31546753 | 15 | 3666 |
| 39. | DSP | chr6:7541906-7585888 | 24 | 9096 |
| 40. | DTNA | chr18:34755967-34890450 | 26 | 3138 |
| 41. | DYSF | chr2:71453989-71686502 | 58 | 7650 |
| 42. | EMD | chrX:154379475-154381207 | 6 | 885 |
| 43. | EYA4 | chr6:133274771-133531236 | 21 | 2477 |
| 44. | FHL1 | chrX:136196793-136210035 | 8 | 1312 |
| 45. | FHL2 | chr2:105361273-105399555 | 10 | 1866 |
| 46. | FHOD3 | chr18:36297826-36779540 | 29 | 5450 |
| 47. | FKRP | chr19:46755441-46756948 | 1 | 1508 |
| 48. | FKTN | chr9:105575023-105640133 | 13 | 1926 |
| 49. | FLNA | chrX:154348839-154371255 | 47 | 8901 |
| 50. | FLNC | chr7:128830628-128858533 | 48 | 9138 |
| 51. | FXN | chr9:69035773-69099944 | 6 | 795 |
| 52. | GAA | chr17:80104577-80119341 | 20 | 3264 |
| 53. | GATA4 | chr8:11708303-11758485 | 6 | 1452 |
| 54. | GATA5 | chr20:62464826-62475531 | 6 | 1314 |
| 55. | GATA6 | chr18:22171135-22200833 | 6 | 1908 |

| | | | | |
|---|---|---|---|---|
| 56. | GATAD1 | chr7:92447720-92456572 | 5 | 910 |
| 57. | GDF1 | chr19:18868587-18870317 | 2 | 1159 |
| 58. | GJA5 | chr1:147758152-147759248 | 1 | 1097 |
| 59. | GLA | chrX:101397799-101407913 | 8 | 1480 |
| 60. | GPD1L | chr3:32106702-32165920 | 8 | 1216 |
| 61. | HAND1 | chr5:154475796-154478018 | 2 | 688 |
| 62. | HCN4 | chr15:73322471-73368280 | 8 | 3772 |
| 63. | HFE | chr6:26087431-26094421 | 8 | 1248 |
| 64. | HRAS | chr11:532626-534332 | 5 | 752 |
| 65. | ILK | chr11:6604262-6610621 | 12 | 1714 |
| 66. | ISPD | chr7:16091685-16421332 | 10 | 1556 |
| 67. | JPH2 | chr20:44114786-44186715 | 6 | 2222 |
| 68. | JUP | chr17:41755734-41771864 | 13 | 2498 |
| 69. | KCNA5 | chr12:5044138-5045999 | 1 | 1862 |
| 70. | KCND3 | chr1:111776067-111982736 | 7 | 2108 |
| 71. | KCNE1 | chr21:34449235-34449644 | 1 | 410 |
| 72. | KCNE2 | chr21:34370469-34370860 | 1 | 392 |
| 73. | KCNE3 | chr11:74457242-74457573 | 1 | 332 |
| 74. | KCNE5 | chrX:109624582-109625030 | 1 | 449 |
| 75. | KCNH2 | chr7:150945355-150977923 | 16 | 4177 |
| 76. | KCNJ2 | chr17:70175030-70176333 | 1 | 1304 |
| 77. | KCNJ5 | chr11:128911264-128916741 | 2 | 1300 |
| 78. | KCNJ8 | chr12:21765713-21773626 | 2 | 1315 |
| 79. | KCNQ1 | chr11:2444676-2848013 | 20 | 2635 |
| 80. | KRAS | chr12:25209785-25245394 | 6 | 828 |
| 81. | LAMA4 | chr6:112109427-112254160 | 40 | 6637 |
| 82. | LAMP2 | chrX:120428474-120469179 | 11 | 1736 |
| 83. | LDB3 | chr10:86668682-86732986 | 16 | 2840 |

| | | | | |
|---|---|---|---|---|
| 84. | LEFTY2 | chr1:225937411-225941150 | 4 | 1201 |
| 85. | LMNA | chr1:156114909-156139849 | 17 | 2729 |
| 86. | LMOD3 | chr3:69109085-69122396 | 3 | 1743 |
| 87. | LRRC10 | chr12:69609995-69610848 | 1 | 854 |
| 88. | LZTR1 | chr22:20982362-20997358 | 22 | 3249 |
| 89. | MAP2K1 | chr15:66387338-66490625 | 12 | 1462 |
| 90. | MAP2K2 | chr19:4090588-4123885 | 11 | 1423 |
| 91. | MIB1 | chr18:21741574-21864676 | 21 | 3441 |
| 92. | MMP21 | chr10:125766652-125775831 | 7 | 1850 |
| 93. | MRAS | chr3:138372874-138402279 | 5 | 727 |
| 94. | MYBPC3 | chr11:47331861-47352657 | 34 | 4515 |
| 95. | MYBPHL | chr1:109294229-109307001 | 8 | 1225 |
| 96. | MYH6 | chr14:23382030-23407233 | 37 | 6560 |
| 97. | MYH7 | chr14:23412844-23433742 | 38 | 6568 |
| 98. | MYL2 | chr12:110911067-110920539 | 7 | 675 |
| 99. | MYL3 | chr3:46858234-46863400 | 6 | 708 |
| 100. | MYL4 | chr17:47209413-47223052 | 6 | 807 |
| 101. | MYLK2 | chr20:31819571-31833807 | 12 | 2031 |
| 102. | MYOF | chr10:93306953-93482204 | 55 | 7397 |
| 103. | MYOM1 | chr18:3067252-3215233 | 37 | 5799 |
| 104. | MYOT | chr5:137870642-137887395 | 9 | 1677 |
| 105. | MYOZ2 | chr4:119136516-119186210 | 5 | 895 |
| 106. | MYPN | chr10:68106715-68210465 | 21 | 4521 |
| 107. | NEBL | chr10:20785737-21173843 | 34 | 4296 |
| 108. | NEXN | chr1:77916097-77942923 | 13 | 2302 |
| 109. | NF1 | chr17:31095198-31378941 | 63 | 10271 |
| 110. | NKX2-5 | chr5:173232559-173235093 | 5 | 1295 |
| 111. | NKX2-6 | chr8:23702441-23706608 | 2 | 946 |
| 112. | NPPA | chr1:11845993-11847694 | 3 | 522 |

| | | | | |
|---|---|---|---|---|
| 113. | NRAS | chr1:114708525-114716170 | 4 | 650 |
| 114. | NUP155 | chr5:37291890-37370987 | 35 | 4884 |
| 115. | PDLIM3 | chr4:185502284-185535444 | 9 | 1463 |
| 116. | PKD1L1 | chr7:47775133-47948450 | 57 | 9726 |
| 117. | PKP2 | chr12:32792414-32896741 | 15 | 2963 |
| 118. | PLEC | chr8:143916167-143975379 | 43 | 15845 |
| 119. | PLEKHM2 | chr1:15684549-15733944 | 20 | 3460 |
| 120. | PLN | chr6:118558912-118559090 | 1 | 179 |
| 121. | PPA2 | chr4:105369715-105474060 | 14 | 1350 |
| 122. | PPP1CB | chr2:28752115-28799313 | 9 | 1363 |
| 123. | PRDM16 | chr1:3069250-3433821 | 18 | 4215 |
| 124. | PRKAG2 | chr7:151557191-151876630 | 19 | 2353 |
| 125. | PSEN1 | chr14:73148010-73219299 | 12 | 1913 |
| 126. | PSEN2 | chr1:226880636-226895589 | 11 | 1666 |
| 127. | PTPN11 | chr12:112419102-112504774 | 16 | 2163 |
| 128. | RAF1 | chr3:12584504-12618731 | 17 | 2347 |
| 129. | RANGRF | chr17:8288779-8289946 | 4 | 733 |
| 130. | RBM20 | chr10:110644445-110835988 | 14 | 3964 |
| 131. | RIT1 | chr1:155900378-155910895 | 7 | 859 |
| 132. | RRAS | chr19:49635566-49640108 | 6 | 777 |
| 133. | RYR2 | chr1:237042512-237832657 | 105 | 17087 |
| 134. | SALL4 | chr20:51784255-51802418 | 4 | 3242 |
| 135. | SCN10A | chr3:38697339-38794020 | 27 | 6411 |
| 136. | SCN1B | chr19:35030811-35039711 | 5 | 1116 |
| 137. | SCN2B | chr11:118166877-118176441 | 4 | 728 |
| 138. | SCN3B | chr11:123634133-123653811 | 5 | 748 |
| 139. | SCN4B | chr11:118137017-118152683 | 5 | 787 |
| 140. | SCN5A | chr3:38550311-38633317 | 29 | 6785 |
| 141. | SCNN1G | chr16:23186262-23215479 | 12 | 2190 |

| | | | | |
|---|---|---|---|---|
| 142. | SDHA | chr5:218346-256430 | 16 | 2449 |
| 143. | SGCD | chr5:156329567-156759400 | 9 | 1182 |
| 144. | SHOC2 | chr10:110964349-111011828 | 8 | 1909 |
| 145. | SLMAP | chr3:57757642-57927398 | 24 | 3263 |
| 146. | SNTA1 | chr20:33408497-33443630 | 8 | 1678 |
| 147. | SOS1 | chr2:38985814-39120432 | 23 | 4462 |
| 148. | SOS2 | chr14:50118334-50231293 | 24 | 4626 |
| 149. | SPEG | chr2:219434968-219498199 | 50 | 11763 |
| 150. | SPRED1 | chr15:38253176-38351674 | 7 | 1475 |
| 151. | SYNE1 | chr6:152122426-152628341 | 154 | 30882 |
| 152. | SYNM | chr15:99105190-99133068 | 4 | 4778 |
| 153. | SYNPO2L | chr10:73646708-73655932 | 5 | 3134 |
| 154. | TAZ | chrX:154411834-154421014 | 10 | 1254 |
| 155. | TBX20 | chr7:35202420-35253630 | 8 | 1508 |
| 156. | TBX5 | chr12:114355522-114403908 | 8 | 1785 |
| 157. | TCAP | chr17:39665350-39666119 | 2 | 544 |
| 158. | TECRL | chr4:64277025-64409361 | 13 | 1371 |
| 159. | TGFB3 | chr14:75959177-75980903 | 7 | 1383 |
| 160. | TMEM43 | chr3:14125184-14141805 | 13 | 1652 |
| 161. | TMPO | chr12:98515858-98547868 | 10 | 3169 |
| 162. | TNNC1 | chr3:52451265-52454025 | 6 | 606 |
| 163. | TNNI3 | chr19:55151824-55157599 | 8 | 876 |
| 164. | TNNI3K | chr1:74235442-74543992 | 28 | 3237 |
| 165. | TNNT2 | chr1:201359200-201373264 | 18 | 1307 |
| 166. | TPM1 | chr15:63042820-63071182 | 17 | 1971 |
| 167. | TRDN | chr6:123218591-123636785 | 44 | 3418 |
| 168. | TRPM4 | chr19:49157857-49211508 | 25 | 4149 |
| 169. | TTN | chr2:178527002-178804652 | 364 | 121875 |
| 170. | TTR | chr18:31591893-31598685 | 8 | 718 |
| 171. | VCL | chr10:73998198-74118179 | 22 | 3845 |
| 172. | ZIC3 | chrX:137566682-137577281 | 4 | 1634 |
